# Supplementary material for: Organization and dynamics of the SpoVAEa protein and its surrounding inner membrane lipids, upon germination of Bacillus subtilis spores
Source: Sci Rep. 2022 Mar 23;12:4944. doi: 10.1038/s41598-022-09147-3 (PMC8943000; doi:10.1038/s41598-022-09147-3)
Supplement: Supplementary file 1 — Supplementary Information. [file 41598_2022_9147_MOESM1_ESM.docx]

**Supplementary information Wen et al. 2022**


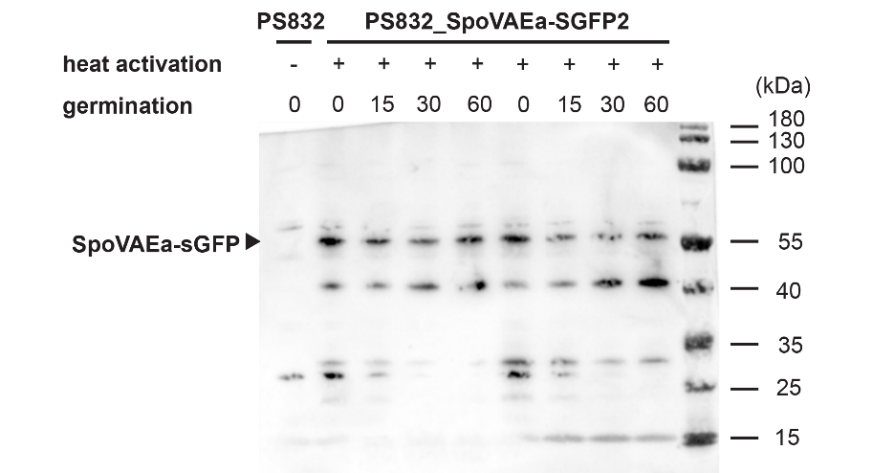


**Figure S1.** Western blot of SpoVAEa-SGFP2 in dormant and germinated PS832 SpoVAEa-SGFP2 spores. Spore germination was induced by (10 mM each) AGFK after a 30 min heat treatment at 70°C to promote and synchronize spore germination. Proteins were extracted from two independent batches of spores germinated for 0, 15, 30, and 60 min, and run on SDS-PAGE (first and second series respectively). Proteins were transferred to a PDVF membrane and SpoVAEa-SGFP2 was detected with polyclonal rabbit anti-GFP antibodies (Abcam) followed by an HRP-conjugated secondary goat anti-rabbit lgG H&L antibody (Abcam). The arrow indicates the expected molecular weight of the fusion protein. As expected, that protein is absent in the wild-type PS832 strain. The increase in the intensities of the 42 and 15 kDa bands at the expense of the 57 kDa band likely reflects some degradation of the fusion protein in this spore sample set. In addition, the SpoVAEa-SGFP2 spores contain some other anti-GFP cross-reactive material that might be additional breakdown products or otherwise cross-reactive proteins differentially expressed possibly due to the SpoVAEa-SGFP2 overexpression.


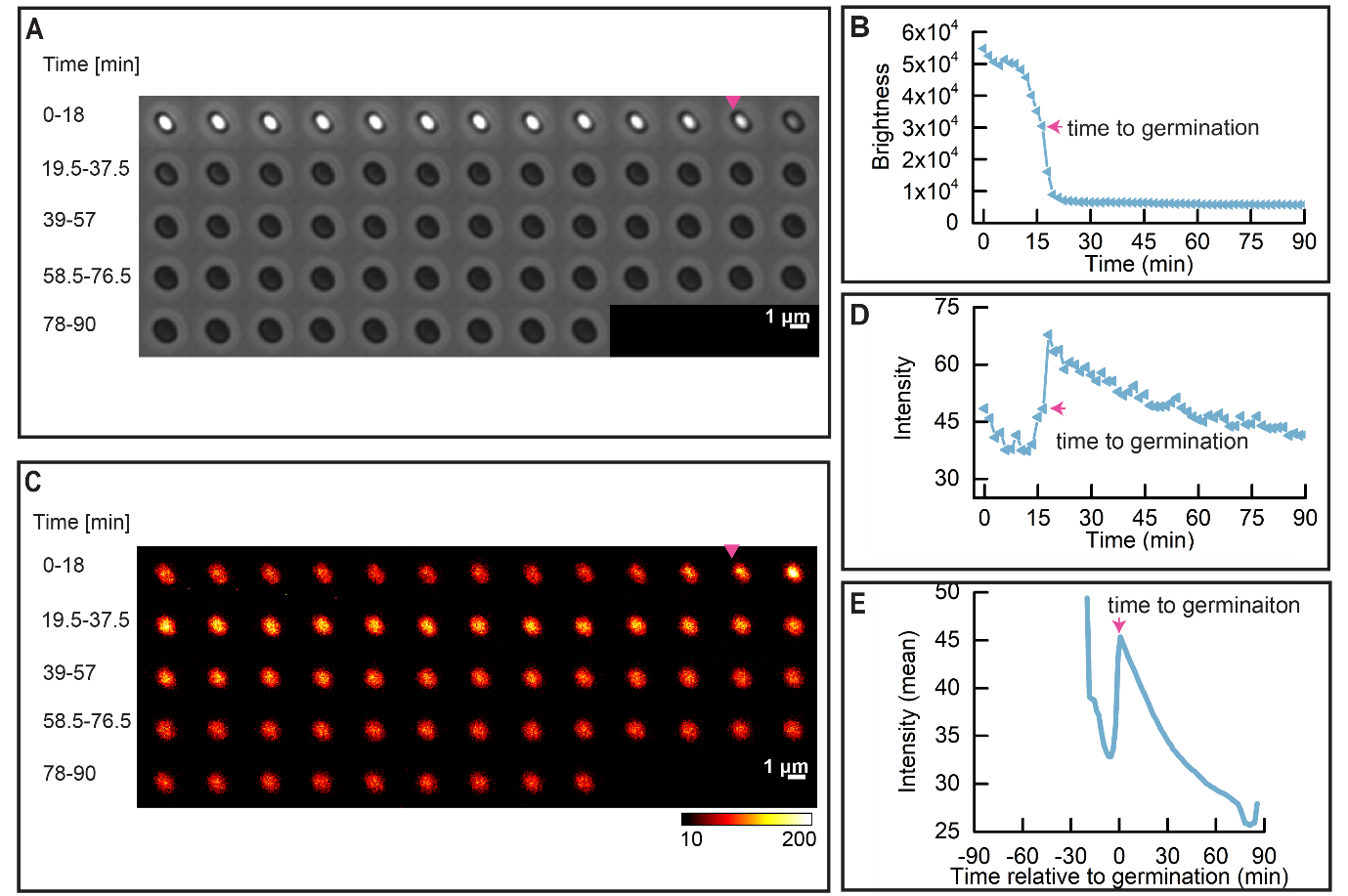


**Figure S2**. Dynamics of SpoVAEa-SGFP2 during heat activated spore germination. Spore germination was triggered by (10 mM each) AGFK after 5 hours of heat activation at 65 °C. (A) Phase contrast time lapse images of a single PS832 SpoVAEa-SGFP2 spore; the magenta arrow indicates the ‘time to germination’. (B) The brightness profile corresponding to the images shown in panel A. (C) The fluorescence time lapse images of the same spore shown in panel A. (D) The SpoVAEa-SGFP2 fluorescence intensity profile corresponding to images in the panel C.; the magenta arrow indicates the ‘time to germination’. (E) Average of 562 synchronized single SpoVAEa-SGFP2 spore fluorescence intensity traces. Synchronization defines t=0 min as the ‘time to germination’. 595 spores’ were tracked by microscopy for 90 min, 94.5% of them completed germination. Notably, the drop in SpoVAEa-SGFP2 fluorescence intensity in E) before the ‘time to germination’ was most likely due to bleaching of the spore’s autofluorescence.


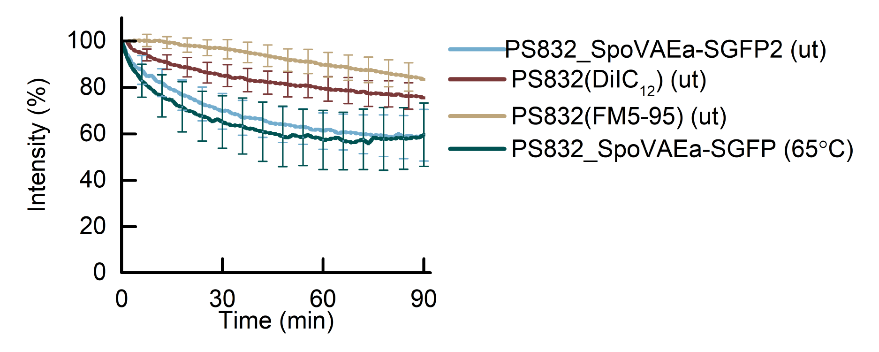


**Figure S3**. Loss of fluorescent intensity of dormant spores tracked by time-lapse imaging. Analyzed dormant spores came from the same populations presented in Fig. 2, 3, 4, and S2, respectively, and these spores didn’t respond to AGFK induced germination in the 90 min time-lapse imaging process. The image acquisition and image analysis were detailed in the Materials and Methods, as well as in legends of corresponding figures mentioned above. The total number of spores examined of strains PS832_SpoVAEa-SGFP2 (ut), PS832(DiIC_12_) (ut), PS832(FM5-95) (ut), and PS832_SpoVAEa-SGFP2 (65°C) spores were 195, 119, 92, and 33, respectively. ut, untreated spores. 65°C, heat treatment at 65°C for 5 hours.

Supplementary Table 1. Pearson correlation between spore brightness and green autofluorescence or fluorescence intensity of SpoVAEa-SGFP2.

| T (°C) | **PS832** | | | **PS832 SpoVAEa-SGFP2** | | | **PS4150** | | | **PS4150 SpoVAEa-SGFP2** | | |
| --- | --- | --- | --- | --- | --- | --- | --- | --- | --- | --- | --- | --- |
|  | No. spores | Pearson correlation | | No. spores | Pearson correlation | | No. spores | Pearson correlation | | No. spores | Pearson correlation | |
|  |  | r | p-value |  | r | p-value |  | r | p-value |  | r | p-value |
| ut | 672 | 0.54 | < 0.01 | 1201 | -0.13 | < 0.01 | 700 | -0.021 | 0.57 | 1287 | 0.0068 | 0.81 |
| 40 | 1577 | 0.56 | < 0.01 | 1057 | 0.036 | 0.25 | 1135 | 0.083 | < 0.01 | 1254 | 0.083 | < 0.01 |
| 50 | 718 | 0.54 | < 0.01 | 948 | 0.1 | < 0.01 | 553 | -0.008 | 0.85 | 1174 | 0.041 | 0.17 |
| 60 | 1207 | 0.28 | < 0.01 | 951 | 0.19 | < 0.01 | 839 | 0.013 | 0.72 | 1511 | 0.033 | 0.20 |
| 65 | 1250 | 0.29 | < 0.01 | 1121 | 0.29 | < 0.01 | 1248 | 0.047 | 0.11 | 1249 | 0.017 | 0.56 |
| 70 | 1016 | 0.47 | < 0.01 | 1123 | 0.35 | < 0.01 | 1377 | 0.047 | 0.079 | 1358 | 0.023 | 0.39 |
| 75 | 816 | 0.32 | < 0.01 | 1133 | 0.32 | < 0.01 | 1541 | -0.18 | < 0.01 | 937 | 0.3 | < 0.01 |
| 80 | 444 | 0.37 | < 0.01 | 1273 | 0.045 | 0.11 | 1967 | 0.53 | < 0.01 | 1109 | 0.7 | < 0.01 |

ut: untreated spores

Supplementary Table 2. Pearson correlation between spore brightness and fluorescence intensity of dye stained IM in spores given different heat treatments.

| T (°C) | **832(DiIC_12_)** | | | **832(FM5-95)** | | |
| --- | --- | --- | --- | --- | --- | --- |
|  | No. spores | Pearson correlation | | No. spores | Pearson correlation | |
|  |  | r | p-value |  | r | p-value |
| ut | 876 | -0.012 | 0.73 | 1739 | 0.17 | < 0.01 |
| 40 | 955 | -0.012 | 0.53 | 1136 | 0.26 | < 0.01 |
| 50 | 1091 | -0.041 | 0.18 | 1383 | 0.2 | < 0.01 |
| 60 | 1335 | -0.044 | 0.11 | 1284 | 0.23 | < 0.01 |
| 65 | 612 | -0.075 | 0.064 | 1350 | 0.29 | < 0.01 |
| 70 | 583 | -0.013 | < 0.01 | 1639 | 0.54 | < 0.01 |
| 75 | 1472 | -0.22 | < 0.01 | 932 | 0.76 | < 0.01 |
| 80 | 1049 | -0.37 | < 0.01 | 2368 | 0.36 | < 0.01 |

ut: untreated spores \
